# Impact of Social Restrictions During COVID-19 on the Aquatic Levels of Antimicrobials and Other Drugs in Delhi

**DOI:** 10.7759/cureus.60835

**Published:** 2024-05-22

**Authors:** Thirumurthy Velpandian, Moksha Laxmi, Ujjalkumar Das, Gayatri Suresh, Arti Kapil, Nabanita Halder

**Affiliations:** 1 High-Precision Bioanalytical Facility (DST-FIST sponsored) Ocular Pharmacology and Pharmacy Division, Dr. Rajendra Prasad Centre for Ophthalmic Sciences, All India Institute of Medical Sciences, New Delhi, New Delhi, IND; 2 Microbiology, All India Institute of Medical Sciences, New Delhi, New Delhi, IND

**Keywords:** covid-19, antimicrobial resistance, drug disposal, landfill, antibiotics, delhi-ncr

## Abstract

The relative contribution of factors responsible for the environmental exposure of active pharmaceutical ingredients (APIs) is of interest for appropriate remedial measures. This study was carried out to evaluate the post-lockdown levels of APIs in water resources, in comparison to our previously published study from 2016. The environmental levels of 28 drugs from different classes were analyzed in surface water (Yamuna River), aquifers, and leachate samples collected from 26 locations in Delhi-NCR using the previously validated liquid chromatography-mass spectrometry (LC-MS/MS) methods. In addition, the prevalence of antimicrobial resistance in coliforms isolated from targeted surface water samples was also studied. This study revealed that more than 90% of APIs, including antibiotics, decreased drastically in both surface water and aquifers compared to our previous data. Selected samples subjected to antimicrobial resistance (AMR) analysis revealed the presence of cephalosporin-resistant coliform bacteria. Tracing cephalosporins in the surface and drain water samples revealed the presence of ceftriaxone in the drain and water samples from Yamuna River. Higher levels of ceftriaxone in landfill leachate were also found, which were found to be associated with coliform resistance and indicate the un-segregated disposal of medical waste into landfills. Social restrictions enforced due to COVID-19 resulted in a drastic decrease in antimicrobials and other APIs in aquatic water resources. Increased ceftriaxone and cephalosporin resistance was seen in coliform from surface water and drain, indicating the possibility of hospital waste and treatment-related drugs entering Yamuna River. Enforcement of the regulations for the safe disposal of antibiotics at hospitals and preliminary disinfection of hospital sewage before its inflow into common drains might help minimize the spread of antibiotic resistance in the environment.

## Introduction

Environmental exposure to active pharmaceutical ingredients (APIs) like antibiotics poses a potential risk to living organisms because of the emergence of antimicrobial drug resistance (AMR), soil microbiota changes, and consequential health effects. It is estimated that in 2019 alone, approximately 4.95 million deaths were attributed to antibiotic resistance, and, that by 2050, AMR will be responsible for up to 10 million deaths worldwide [1]. Our previous study, conducted in July 2016, reported high levels of APIs, including antibiotics, in surface and groundwater sources in Delhi and the National Capital Region (NCR), which was primarily attributed to un-segregated disposal of waste [2]. The impact of the presence of antibiotics in the aquatic environment has not yet been understood clearly. Since their rate of entry into the environment is higher than the rate of their elimination, antibiotics are considered to be persistent or “pseudo-persistent” environmental pollutants [3]. Biological processes (e.g., bacterial or fungal degradation), and abiotic mechanisms (e.g., oxidation, photolysis, and reduction) can cause the degradation of antibiotics in the environment; however, these are heavily dependent on environmental and physicochemical conditions [4]. Antibiotic degradation products or metabolites generated via these processes can be further transformed into bioactive compounds, which may have higher stability, mobility, and concentrations than parent compounds [5].

Approximately 40-90 % of the dosed antibiotics are excreted unmetabolized, in the active form of urine or feces. Hence, one of the critical elements in preventing ecological contamination is to reduce the unwarranted use of antibiotics in agriculture fields and livestock farms [6]. However, the relative contribution of factors responsible for the environmental exposure of antibiotics and other APIs is not conclusive. In addition, the analysis of environmental drug disposal patterns remains a challenge for calculating the contribution of drugs eliminated from the body after consumption and from unutilized drug disposal. However, our previous study highlighted the need for a regulatory framework regarding the disposal of unutilized drugs, the implementation of any such effort is yet to see its day till the completion of this study.

The novel coronavirus disease 2019 (COVID-19), caused by SARS-CoV-2, was recognized by the World Health Organization as a global pandemic on March 11, 2020. Since December 2019, an exponential increase in COVID-19 infection has caused millions of deaths globally. Throughout the world, strict regulations were enforced. In the absence of approved therapeutic modalities, governments throughout the world ordered strict restrictions on the movement of civilians, excluding healthcare workers and lifecare public services, to limit the spread of the disease. The Government of India implemented the lockdown and movement restrictions from March 2020 to September 2021 in various phases. Interestingly, these actions had a beneficial impact on several environmental parameters in water resources [7].

Therefore, the imposition of the lockdown during the COVID-19 pandemic presented a rare opportunity to test the environmental levels of APIs in water resources in a scenario of restricted anthropogenic activities. In continuation to our earlier study published in 2018 [2], this study evaluated the impact of social restrictions on the environmental levels of antimicrobials and other APIs. Furthermore, AMR was analyzed in aquatic resources in Delhi-NCR (India).

## Materials and methods

Chemicals and reagents

Antifungal agents, such as clotrimazole, itraconazole, ketoconazole, fluconazole, miconazole, voriconazole, and terbinafine; antiprotozoal agents, like tinidazole and metronidazole; and antibacterial agents belonging to the class of β-lactams (amoxicillin), macrolides (azithromycin and erythromycin), fluoroquinolones (ciprofloxacin, moxifloxacin, norfloxacin, ofloxacin, and sparfloxacin), and aminoglycosides (amikacin, kanamycin, gentamicin, neomycin, netilmicin, tobramycin, and streptomycin) were subjected for analysis in this study. Diclofenac and ibuprofen were selected to represent highly used drugs from the group of nonsteroidal anti-inflammatory drugs (NSAIDs). Cetirizine and amlodipine were selected from the group of anti-cold medication (H1 receptor blocker) and anti-hypertensive (calcium channel blocker), respectively, for the analysis, as mentioned in an earlier study from our laboratory [2]. The pharmaceutical standards were obtained from various commercial sources, namely, Medleys Pharmaceuticals (Mumbai, India), French Capital Pharmaceuticals (Baddi, HP, India), Helios Pharmaceuticals (Baddi, HP, India), Microlab (Chennai, India), and ZIM Labs (Nagpur, MP, India). Sulfadimethoxine (SDM) was purchased from Sigma-Aldrich (St. Louis, MO, USA). Liquid chromatography-mass spectrometry (LC-MS)-grade formic acid (FA), acetonitrile, and methanol were procured from Merck (Darmstadt, Germany). Fresh, ultrapure water of 18.2 MΩ resistance from a Milli-Q Gradient system (Millipore Corp., Bedford, MA, USA) was used for the study. All chemicals and solvents used were of the highest analytical grades available.

Instrumentation and analysis

Liquid chromatography coupled electrospray tandem mass spectrometry (LC-ESI-MS/MS) experiments were performed using a triple quadrupole tandem mass spectrometer (4000 Q-Trap, AB Sciex, Foster City, CA, USA) coupled with a high-performance liquid chromatography system (HPLC, Agilent Technologies, 1260 Infinity, Santa Clara, CA, USA) consisted of a quaternary pump (G1311C), multisampler (G7167A), thermostatted column compartment (G1316A) with a variable-wavelength UV detector (G1314F), and online degasser. All the parameters of the tandem mass spectrometer and HPLC were controlled by the Analyst software, version 1.7.1 (AB Sciex, Foster City, CA, USA) and OpenLAB control panel software (Agilent Technologies, 1260 Infinity, Santa Clara, CA, USA), respectively.

Sample collection

**Figure 1:**
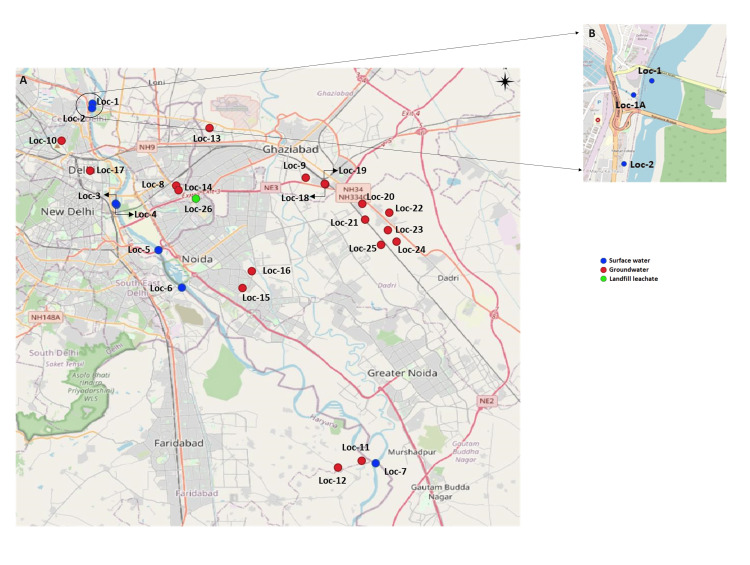
A) Locations of Delhi-NCR for the estimation of antimicrobials and other APIs in surface and aquifers samples for five years (2016-2021) (plotted using QGIS, version 3.18). B) Locations of Delhi-NCR selected for the microbiological analyses.

Samples were collected in May 2021, which is the summer season in Delhi-NCR, prior to the monsoon. Surface water samples (500 mL) from seven locations of Yamuna River in Delhi-NCR (radius of 50 km) were collected in sterile glass bottles at various access points correlating with the previously conducted study in 2016. Water samples (grab samples) were collected per WHO guidelines for water collection [8]. Groundwater samples were collected directly from the hand-operated or motorized water pumps that were connected to the borewells, in glass bottles, as a midstream collection after allowing them to run for a few minutes. These samples were collected from areas that were accessible and permitted by the owners for analysis. Groundwater samples (500 mL) were collected from 18 locations (40-150 ft depth). The single landfill leachate sample was collected directly from the oozing leachate draining from the landfill in glass bottles, from an accessible area adjacent to the landfill. The locations for sample collection correlated with those from our previous study conducted in 2016 [2], and the exact locations of sample collection were plotted using QGIS, version 3.18 (QGIS Geographic Information System, QGIS Association) (Figure 1 and Table 1).

**Table 1:** List of sampling sites along with coordinates for the collection of surface, aquifer water samples, and leachate within the radius of 50 km around Delhi-NCR. Loc: location

Surface water sampling sites (n = 7)			
Name	Latitude	Longitude	Locations
Loc 1	28°42'44.14"N	77°13'53.94"E	Sur Ghat (from the origin)
Loc 2	28°42'29.52"N	77°13'52.26"E	Sur Ghat (after shutter)
Loc 3	28°37'12.32"N	77°15'12.63"E	Nala
Loc 4	28°37'15.87"N	77°15'9.86"E	Yamuna before mixing Nala
Loc 5	28°34'43.40"N	77°17'30.25"E	Mayur Vihar (flyover)
Loc 6	28°32'40.30"N	77°18'47.17"E	Sarita Vihar (flyover)
Loc 7	28°23'3.52"N	77°29'22.97"E	Yamuna water II (with Datura Plant)
Aquifer water samples sites (n = 18)			
Loc 8	28°38'15.03"N	77°18'28.57"E	Patparganj industrial area (Invictus)
Loc 9	28°38'41.10"N	77°25'33.62"E	Vijay Nagar, Ghaziabad, UP
Loc 10	28°40'42.48"N	77°12'12.27"E	Shora Kothi, Ghantaghar
Loc 11	28°23'11.61"N	77°28'37.07"E	Chedesi
Loc 12	28°22'49.50"N	77°27'19.37"E	Manjhavali
Loc 13	28°41'24.06"N	77°20'17.83"E	Shalimar Garden (Sahibabad)
Loc 14	28°38'0.00"N	77°18'36.09"E	Patpadganz handpump (Near Max Hospital)
Loc 15	28°32'38.89"N	77°22'6.00"E	Noida sector 100, Borewell water
Loc 16	28°33'34.23"N	77°22'36.38"E	Noida sector 49
Loc 17	28°39'4.07"N	77°13'46.05"E	Chandni Chowk (handpump water)
Loc 18	28°38'19.46"N	77°26'37.55"E	Sri Guru Kripa Industries, submersible pump)
Loc 19	28°38'21.23"N	77°26'35.86"E	Near Suruchi Dyeing Udyog (120 ft)- HP water
Loc 20	28°37'15.40"N	77°28'39.13"E	Surya Processor Pvt Ltd (HP water)
Loc 21	28°36'23.50"N	77°28'48.38"E	Khera Dharampura (HP water)
Loc 22	28°36'46.18"N	77°30'7.65"E	Residential area, Bishnoli (HP water)
Loc 23	28°35'48.89"N	77°30'3.14"E	Shiv Mandir, Achheja (HP water)
Loc 24	28°35'11.21"N	77°30'31.53"E	Sadopur Gram
Loc 25	28°35'0.69"N	77°29'40.59"E	Sadullapur Village
Leachate sample (n = 1)			
Loc 26	28°62'56.49"N	77°32'58.46"E	Gazipur Landfill

Characterization and storage of collected samples

The collected samples were brought to the laboratory on the same day and centrifuged at 7000 g for 10 minutes at room temperature. Following this, the samples were aliquoted in triplicates and analyzed for osmolarity, total dissolved solids (TDS), and pH. Osmolarity was quantified using the principle of freezing point depression by placing 50 μL of water sample in the sample holder, which was analyzed using a calibrated osmometer (μOsmette, Precision Systems, USA) [2]. TDS in the water samples was assessed using a TDS meter (TDS-3, Korea). Similarly, a calibrated pH meter (pH 510, Cyberscan, Thermo Scientific, USA) was used to analyze the pH of the water samples. The individual value was derived by taking the average of three readings for all the samples. Following this, an aliquot of 10 mL of each sample in duplicate was placed into 20 mL borosilicate vials, then lyophilized, and stored at −80 °C until analysis.

Sample preparation for analysis

For the analysis, the lyophilized water samples were reconstituted in 1 mL of 50 % acetonitrile in water and vortexed for 5 min. An aliquot of 100 μL from a reconstituted vial was mixed with 100 μL of the solution containing sulfadimethoxine (IS) at a concentration of 25 ng/mL in acetonitrile/water (1:1) with 0.1 % formic acid and subjected to centrifugation at 7000 g for 10 minutes. The resulting clear supernatant (150 μL) was subjected to API quantification using the LC-MS/MS methods below. Similarly, for the leachate analysis, 100 μL of the landfill leachate sample was added to 100 μL of 50% acetonitrile with 0.1% formic acid in water containing 25 ng/mL sulfadimethoxine (IS), vortexed, and centrifuged at 7800 g for 10 minutes. The clear supernatant (100 μL) was subjected to LC-MS/MS analysis. A mixture of APC standards spiked in 1 mL of acetonitrile/water (1:1) was serially diluted to lower concentrations and analyzed to obtain the calibration curve [2].

ESI-LC-MS/MS conditions for groups 1, 2, and 3

For the separation of all the antimicrobials and other active pharmaceutical compounds in group 1 (clotrimazole, itraconazole, ketoconazole, fluconazole, metronidazole, miconazole, terbinafine, tinidazole, and voriconazole), group 2 (amoxicillin, ciprofloxacin, ofloxacin, azithromycin, erythromycin, moxifloxacin, norfloxacin, sparfloxacin, diclofenac, ibuprofen, cetirizine, and amlodipine), and group 3 (amikacin, kanamycin, gentamicin, tobramycin, neomycin, netilmicin, and streptomycin), previously reported methods were followed [2].

Testing for AMR in coliform bacteria isolated from the surface water sample

Three surface water samples were chosen for a pilot study to analyze the prevalence of antibiotic resistance in coliforms. The samples were collected from the entry of Yamuna River into Delhi (Location 1), a major drain before meeting the river (Locatio 1A) (Najafgarh drain), and the place of the river exit from Delhi (Location 27) (Figure 1). The samples were filtered aseptically using Whatman paper to remove larger particles, plated onto MacConkey agar, and incubated overnight at 37°C. After incubation, pink colonies (indicative of coliforms) were sub-cultured on a MacConkey agar until a pure culture with isolated colonies was obtained.

The study of antimicrobial resistance was carried out via the disc diffusion method. For each antibiotic to be tested, in-house antibiotic discs were prepared aseptically to give the final concentration as authorized by the Clinical and Laboratory Standards Institute (CLSI). Control discs were prepared by loading the filter paper discs with water or methanol and dried. For the disc diffusion protocol, a single colony from the pure coliform culture was inoculated in Luria-Bertani broth at 37°C till an OD600 = 0.08 was achieved. Following this, the culture was inoculated on nutrient agar plates using sterile cotton swabs, and the prepared antibiotic discs were placed on the agar surface aseptically using sterile forceps. Plates were incubated overnight in an upright position at 37°C, after which the zone of clearance around each disc was measured. The experiment was carried out in duplicates, and the sensitivity or resistance of the isolate against the particular antibiotic was determined according to the breakpoints set by the CLSI.

Quantification of cephalosporins in selected water samples

The water samples used for the assessment of AMR were selected for the quantification of 18 cephalosporins, namely, cefoxitin, cephalothin, cefuroxime, ceftaroline, cefdinir, cefalexin, cefradine, cefixime, cefaclor, cefotaxime, cefadroxil, cefazolin, ceftriaxone, cefsulodin, cefoperazone, ceftazidime, cefepime, and ceftiofur, using LC-MS/MS. The instrumentation parameters are given in the supplementary file (see Appendix).

Statistical analyses

Statistical analyses for the obtained data were carried out using paired t-test (GraphPad Prism, version 8, Boston, Massachusetts, USA), and a value of p < 0.05 was considered significant.

## Results

Water collection and the assessment of pH, dissolved solutes, and osmolarity

For this study, seven Yamuna River water samples, 18 borewell (aquifers) samples, and one landfill leachate sample were collected from the same sites (as per the GPS location), as our previous study published in 2018. The present study observed, at the mean level, a 2% increase in pH, a 15% decrease in TDS, and an 87% decrease in osmolarity in the surface water (p = 0.58, 0.11, and <0.001, respectively). A mean increase of 1% in pH, 63% increase in TDS, and 27% decrease in osmolarity were observed in aquifers (p = 0.19, 0.16, and 0.7, respectively).

Effect of social restriction on the levels of antimicrobials and other APIs in surface water and aquifers

**Figure 2:**
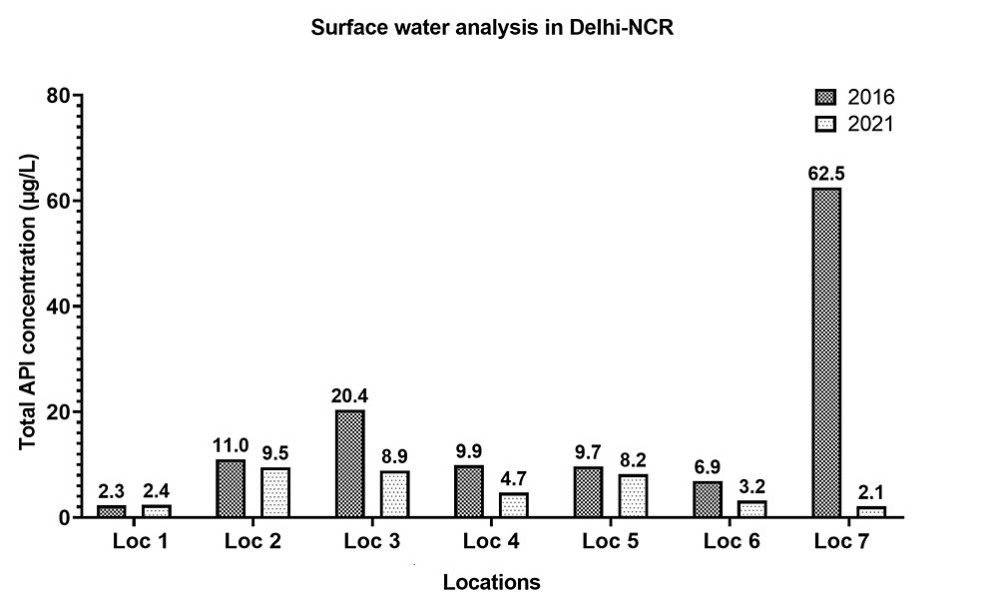
Altered levels of antimicrobials and other APIs in surface samples at different locations of Delhi-NCR over five years (2016-2021)

**Figure 3:**
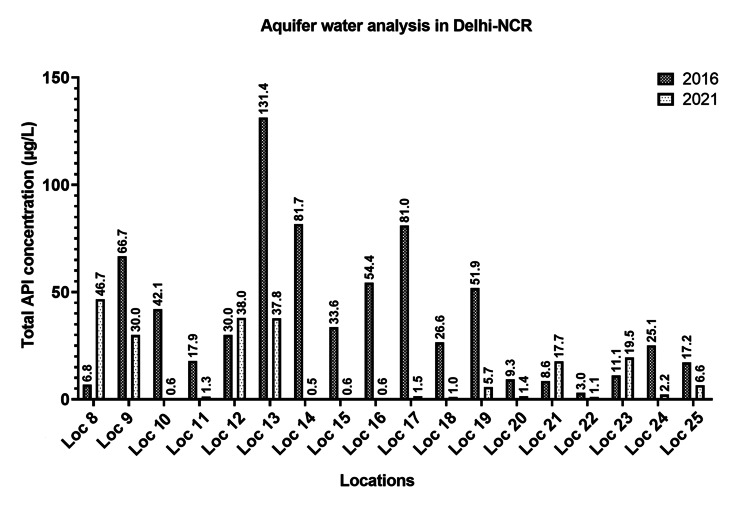
Altered levels of antimicrobials and other APIs in aquifer samples at different locations of Delhi-NCR over five years (2016-2021)

The water samples (surface and aquifers, n = 25) were assessed for the levels of antifungals, fluoroquinolones, β-lactams, aminoglycosides, antiprotozoals, commonly used agents such as non-steroidal anti-inflammatory agents, anti-cold medication, and an anti-hypertensive agent. A noteworthy decrease in the levels (µg/L) of the majority of studied antimicrobials and APIs in the surface and aquifer water samples was observed in this study, compared to the study conducted five years earlier, as shown in Figure 2 and Figure 3, respectively.

**Figure 4:**
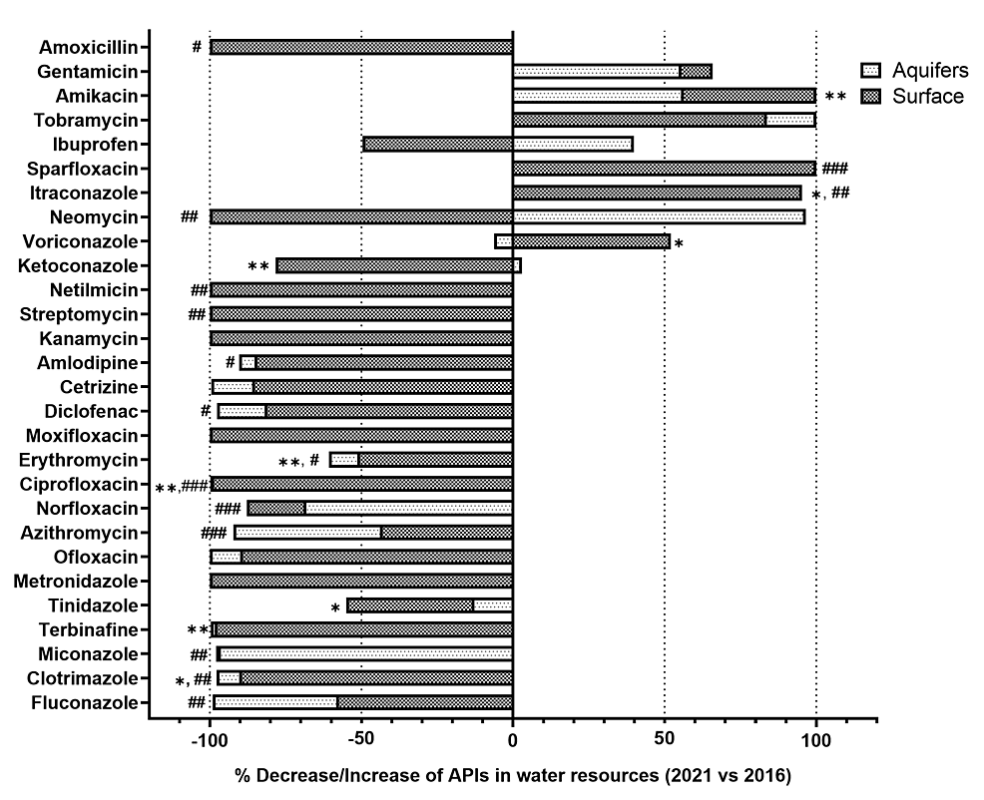
Altered percentage (increase/decrease) in the median levels of 28 antimicrobials and other APIs in all water samples (surface and aquifers) over five years (2016-2021). * p < 0.05, ** p < 0.01, *** p < 0.001 for surface water; and # p < 0.05, ## p < 0.01, ### p < 0.001 for aquifer water samples

On the other hand, the levels of itraconazole, tobramycin, amikacin, gentamicin, neomycin, and ibuprofen were increased in all water samples (n = 25) while comparing it with the levels from our previous study (2016). In comparison to the quantified levels in 2016, the overall altered percentage increase/decrease in the mean levels of 28 antimicrobials and other APIs in surface and aquifer water samples are shown in Figure 4.

In the surface water samples, a significant decrease was observed in the levels of ketoconazole (p = 0.0090), clotrimazole (p = 0.01), terbinafine (p = 0.003), tinidazole (p = 0.041), ciprofloxacin (p = 0.005), and erythromycin (p = 0.004) in 2021 as compared to 2016. In addition, a few APIs like voriconazole (p = 0.036), itraconazole (p = 0.029), and amikacin (p = 0.002) were significantly increased as compared to our previous study.

In aquifer water samples, a significant decrease in the levels of fluconazole (p = 0.003), clotrimazole (p = 0.009), miconazole (p = 0.004), amlodipine (p = 0.022), azithromycin (p < 0.001), norfloxacin (p < 0.001), ciprofloxacin (p < 0.001), amoxicillin (p = 0.013), diclofenac (p = 0.023), streptomycin (p = 0.002), erythromycin (p = 0.032), and netilmicin (p = 0.009) was observed in 2021 as compared to 2016. Few APIs like itraconazole (p = 0.002), sparfloxacin (p < 0.001), and neomycin (p = 0.009) were found to be increased significantly compared to our previously published study.

Effect of social restriction on landfill leachate and localized levels

**Figure 5:**
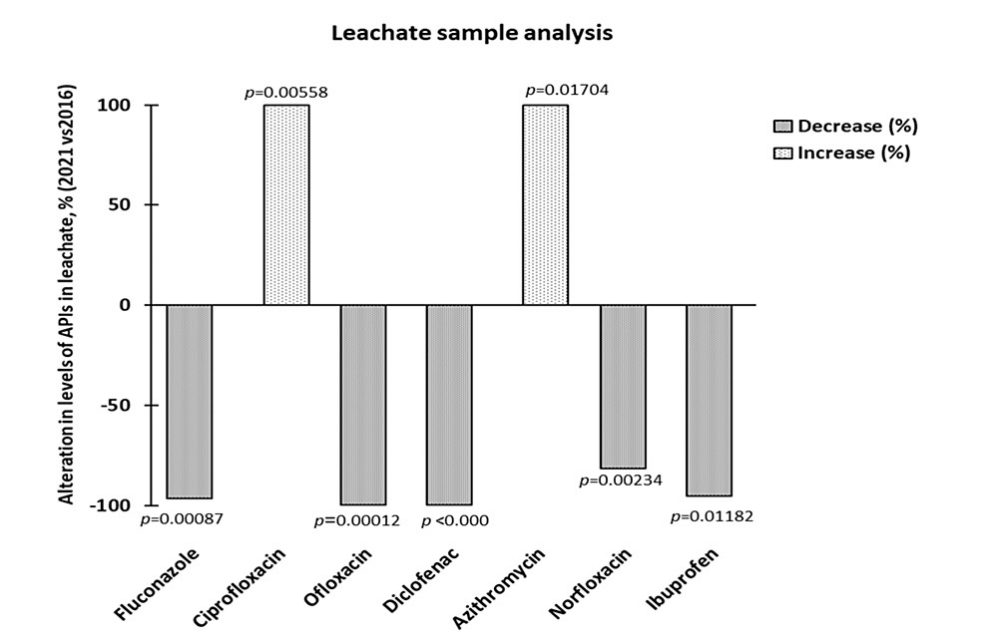
Percentage change in the levels of antimicrobials and other active pharmaceutical ingredients (APIs) in the leachate sample for five years (2016 vs. 2021)

The present study observed the effect of social restriction on the levels of predominantly found APIs in the Ghazipur landfill leachate sample. The results are shown in Figure 5.

A decrease of >95% in the levels of fluconazole, ofloxacin, diclofenac, and ibuprofen was observed in 2021 as compared to the levels in 2016. On the contrary, an increase of >95% was found in the levels of azithromycin and ciprofloxacin in 2021 compared to the levels in 2016. Interestingly, azithromycin, commonly prescribed during the COVID-19 pandemic, was found in leachate samples in 2021 at a concentration of 0.168 µg/L. 

Effect of the environmental levels of antimicrobials on drug resistance

This study revealed the highest prevalence of β-lactam resistance (penicillin and carbapenems) and quinolone resistance in coliform bacteria isolated from Yamuna River. The antibiotic susceptibility results in coliform isolated from Yamuna River showed low-intermediate resistance against aminoglycosides and quinolones, as shown in Table 2.

**Table 2:** Antibiotic susceptibility of coliforms isolated from Yamuna River (Iso-Loc1) and Najafgarh Drain (Iso-Loc1A) R: resistant, S: sensitive, I: intermediate

Antibiotics		Iso-Loc1	Iso-Loc1A
Penicillins	Amoxicillin	R	R
	Piperacillin	I	R
Cephalosporins	Cephalothin	R	R
	Cefixime	R	R
	Cefoperazone	I	R
	Ceftazidime	R	R
	Ceftriaxone	R	R
Quinolones	Ciprofloxacin	I	I
	Gemifloxacin	S	S
	Levofloxacin	S	S
	Lomefloxacin	S	I
	Norfloxacin	S	S
	Ofloxacin	S	I
Macrolides	Azithromycin	R	R
	Clarithromycin	S	R
	Roxithromycin	S	R
Carbapenems	Imipenem	I	R
	Meropenem	S	R
Aminoglycosides	Gentamicin	S	S
	Tobramycin	S	S
	Amikacin	S	S
	Kanamycin	S	I
	Streptomycin	S	R
Polymyxins	Colistin	R	R
Amphenicols	Chloramphenicol	S	S
Oxazolidinones	Linezolid	S	R

The isolates were found to be sensitive (58% and 45%) at Location 1 (Iso-Loc1: isolate from the entry of Yamuna River into Delhi) and Location 1A (Iso-Loc1A: isolate from the Najafgarh Drain before meeting Yamuna River), respectively. The isolates were resistant (27% and 44%) in Iso-Loc1 and Iso-Loc1A, respectively.

Cephalosporin resistance and drug levels

Due to the prevalence of cephalosporin resistance in coliform isolates, surface water and leachate samples were further analyzed to detect the presence of cephalosporins. Among the 18 cephalosporin antibiotics analyzed in the selected surface water samples at Locations 1 and 1A, ceftriaxone was found at a concentration of 0.45 and 0.79 µg/L, respectively. In the leachate sample, the levels of ceftriaxone and cefixime were 23.5 and 2.85 µg/L, respectively.

## Discussion

Antibiotic pollution of water resources has been a point of concern for regulatory agencies due to the potential risk to human health. In India, Yamuna River is considered to be one of the most polluted rivers, as it receives more than 3000 MLD of sewage via several drains. Najafgarh Drain, which is the largest drain in Delhi carries extensive amounts of untreated sewage from hospitals and residential and industrial areas and is potentially one of the major drains responsible for the extensive source of drugs and AMR-related pollution in Yamuna River.

Several agencies are working on developing action plans to address AMR globally and at the national level to tackle this situation. Collective emphasis has been given to understanding the pattern of inappropriate handling of antibiotics and enforcing appropriate policies to reduce drug resistance. The WHO announced AMR as an “urgently high-priority area” and has started a Global Action Plan (GAP) for its containment. Two critical aspects of GAP are strengthening the knowledge through surveillance and research, and the engagement of appropriate legislative action to regulate environmental exposure of antibiotics [9].

Previously, several authors have reported high levels of antibiotic residues in Indian rivers. Mutiyar and Mittal studied the presence and seasonal variation of six targeted antibiotics (ampicillin, ciprofloxacin, gatifloxacin, sparfloxacin, and cefuroxime) in Yamuna River. The highest concentration was observed for ampicillin (13.75 µg L^-1^), and the maximum antibiotic concentrations were observed in winters, followed by summers and monsoons [10]. A study carried out by Biswas and Vellanki reported the presence and spatiotemporal variations of emerging contaminants, such as APIs, personal care products, and endocrine disruptors, across various sites in Yamuna River. Antibiotics present in high concentration, e.g., trimethoprim (8.80 µg L^-1^), ciprofloxacin (0.26 µg L^-1^), enrofloxacin (0.059 µg L^-1^), sulfamethoxazole (1.31 µg L^-1^) also had a high frequency of detection (>90%) [11]. An independent study conducted by Toxics Link (an environmental Non-Governmental Organization) detected the presence of ofloxacin (0.54-0.71 µg L^-1^) and sulfamethoxazole (0.2 µg L^-1^) in Yamuna water samples [12]. This presence of antibiotics in Yamuna River, which is a source of water for drinking and irrigation, is a serious public health concern.

The most significant factors contributing to the entry of antimicrobials in water resources are the discharge of improperly treated effluent from wastewater treatment plants, improper and unsegregated disposal of expired or unused antibiotics, and the overuse/misuse of antimicrobials in humans, animals, or agriculture [2,13]. A decrease in anthropogenic activities is expected to reveal the relative contribution of human usage toward the environmental dissemination of antimicrobial agents. Hence, the COVID-19-related lockdown provided an extremely rare opportunity to evaluate the effect of restricted human movement and activity on the dissemination of antibiotic residues and antibiotic resistance in the environment.

In India, the first nationwide lockdown was implemented in March 2020, extended by three phases, followed by unlocking in more than five phases until the end of 2020. However, due to the re-emergence of COVID-19, a complete lockdown was reinstated from February 2021 till June 2021. Across these 15 months, enforcement agencies heavily restricted anthropogenic activities. In India, retail and recreation, grocery and pharmacy, visits to parks, transit stations, and workplaces, and mobility dropped by 73.4%, 51.2%, 46.3%, 66%, and 56.7%, respectively. On the other hand, mobility to residential places increased by 23.8%, and most people stayed home during the lockdown [14]. Furthermore, Google mobile-based Community Mobility Reports showed that Indian national-level mobility decreased from -38% to -77% for all areas. However, residential mobility showed an increase of 24.6% during the lockdown compared to the reference period [15]. These studies substantially indicated the decrease in anthropogenic activities in India. These studies substantially indicated the decrease in anthropogenic activities in India. This decrease was accompanied by the drastic reduction of pollution in various natural resources.

In the present work, a comparison of API levels in surface and groundwater samples during the premonsoon season between 2016 and 2021 was performed. The current study revealed that more than 90% of the APIs, including antibiotics, decreased drastically during the lockdown in both surface water and aquifers. The levels of the studied antimicrobials and other APIs increased drastically at Loc-3 compared to Loc-1 (entry of Yamuna River into Delhi) due to the entry of Sahibi River, which is recognized as a major Najafgarh drain (Loc-1A). Throughout Yamuna River, the levels were increased, when compared to their corresponding levels observed at Loc-1. The entry of various drains into the Yamuna River carrying human waste could contribute to the increased levels. The movement of antibiotics in monsoons can also lead to the variation of API levels in rivers. However, since the water sampling for this study was carried out during summer (pre-monsoon), the contribution of rainwater in fetching surface APIs into the drain can be excluded [16]. Therefore, human usage could have been the main factor for the levels found in drains. In this study, drug metabolites and their degradation products were not assessed. However, various environmental degradation pathways have already been discussed previously in the literature for some of the drugs studied [3,17,18], and separate studies need to be conducted to comment on their levels while considering the dynamic changes of the surface and aquafer hydro-currents.

Leachate entering into Yamuna River from unscientific landfills can be considered another drain. In our previous study, API levels at Yamuna River surface water exiting from Delhi were very high [2]. These increased levels were predominantly due to the entry of another river tributary into Yamuna River before the last sampling location of water collection in this study. Of note, the levels of studied APIs were multiple-folds lower after the enforcement of social movement restrictions. Therefore, the alteration in the levels of measured APIs during the course of the river in the city of Delhi can only be explained by the entry of river water into the hydrological cycle entering aquifers.

Furthermore, this study observed a significant increase in the levels of ciprofloxacin and azithromycin in the landfill leachate compared to our previous study. During the COVID-19 pandemic, extensive blanket prescription of antibiotics like azithromycin, doxycycline, antiparasitic drug ivermectin, antiviral remdesivir, and zinc supplements has been reported [19,20]. The extensive use of these APIs explains the plausible increased levels of azithromycin and indicates the unsegregated disposal of azithromycin into the landfill. This observation supports our previous finding regarding the unsegregated disposal of antibiotics in landfills [2].

The microbiological analysis was carried out as a pilot study to understand the prevalence and cause of AMR. Considering the extensive AMR observed in the coliform bacteria for cephalosporins, the surface water samples were reanalyzed to quantify the levels of all possible cephalosporins meant for human use. The analysis revealed that ceftriaxone is the only cephalosporin found at a high concentration in surface water and leachate. This finding suggests the association between the extensive ceftriaxone usage and related environmental exposure accompanied by the drug resistance in coliform isolates. As a result, coliform bacteria such as *E. coli* and *Klebsiella* are listed under the critical category in the WHO AMR priority nosocomial pathogens list [21].

Most antibiotics are not completely metabolized in the body and eventually find their way into sewage through urine and feces, contributing to the potential source of fecal coliform bacteria. Another primary source of antibiotics in sewage is the improper disposal of unused antibiotics. Even at low concentrations, the presence of antibiotics in the environment exerts a selection pressure for the development of antibiotic resistance [22]. Sewage is a major environmental reservoir for antimicrobial-resistant genes [23]. Untreated hospital waste draining into sewage is another potential source for transferring resistant microbes into sewage [24].

Since wastewater treatment plants function as a confluence point for bacteria and sewage from various sources, they can be hotspots for selecting and disseminating antibiotic resistance genes [25]. In addition, the high bacterial density in raw sewage provides a favorable environment for the horizontal transfer of antibiotic-resistant genes between bacteria via mobile genetic elements, such as plasmids or transposons [23,26].

A recent study identified a drastic increase in the consumption of fluoroquinolones and third-generation cephalosporins in North Africa, the Middle East, and South Asia [27].

Hospital-based exit interviews and community studies in India indicate that curative injections were prescribed for fever/cough/diarrhea (88.6%) with the opinion that injections are being given for psychological relief to the patients as they insisted on injections [28]. A hospital-based prospective observational study to audit the prescription practices and outcomes in pediatric patients with acute diarrhea revealed that ceftriaxone was the most commonly used injectable antibiotic [29]. Unlike oral antibiotics, injectables like ceftriaxone are used by healthcare professionals in clinics or hospitals where the angle of self-administration may not be associated.

Ceftriaxone levels found in the surface water sample in this study can be attributed to its therapeutic use as it requires parenteral administration by a trained medical professional. Therefore, the resulting AMR in coliform bacteria could also occur due to its therapeutic use. Over or inappropriate use of antibiotics, poor patient compliance, inadequate dosing, or improper choice can increase antibiotic drug resistance in microorganisms [30].

While our study reported a significant decrease in antimicrobial drug levels in the surface water resources and aquifers, higher levels of injectable (ceftriaxone) antibiotics were observed at landfill leachate and sewage drains entering Yamuna River. This finding indicates the lack of appropriate disposal of hospital waste and healthcare professional-related use of antibiotics during the restricted human movement due to the COVID-19 lockdown. However, the AMR observed in coliform bacteria in this study from the sewage and river water sample indicates that the source of such AMR could also be from hospitals, apart from environmentally associated horizontal gene transfer.

The lockdown announced during the COVID-19 pandemic provided us with an unusual opportunity to evaluate API contamination in aquatic resources. While this study supports the concept of wastewater as a reservoir for antimicrobial agents, antimicrobial drug resistance, and antibiotic resistance genes in the environment reported by other studies [21], the drawback is the lack of similar data during the lockdown for comparison. Additional studies are underway in our laboratory to evaluate the API levels in these locations once the lockdown was lifted. These data would provide a more comprehensive view of the effect of anthropological activities on the API levels in water resources.

## Conclusions

To conclude, this study showed that the unexpected social restriction enforced due to COVID-19 resulted in a drastic decrease in antimicrobials and other API levels in the aquatic resources of Delhi-NCR (India). Increased ceftriaxone and cephalosporin resistance in coliform bacteria from surface water and drain indicate the possibility of hospital waste entering into the Yamuna River. Hence, it is imperative to establish a preliminary disinfection process of the hospital sewage before its inflow into the sewage system, which could minimize the spreading of antibiotic-resistant bacteria in the environment.

Professional use of injectable antibiotics like ceftriaxone at hospitals/clinics ultimately leads to their presence in sewage in an unmetabolized form. The main Najafgarh drain (Sahibi River from other states), which eventually joins the Yamuna River receives effluents from several wastewater and sewage treatment plants, which are not equipped for the complete removal of antibiotics and their residues. Therefore, research needs to be targeted toward developing efficient systems (e.g., advanced oxidation processes and bio-remediation) that can be combined with conventional treatment plants to reduce the level of antibiotics reaching the aquatic resources. 

The measurable levels of ceftriaxone observed in the landfill leachate indicate the unsegregated disposal of used, unused, expired drugs from hospitals and clinics, which eventually reach the garbage dump (landfills). The findings from this study could help in the enforcement of regulations on the safe disposal of used/expired antibiotics at hospitals. Focus should also be given toward the designing and implementation of scientific landfills with an impenetrable base layer, which would prevent the contamination of groundwater resources.

Finally, continuous surveillance over longer time durations is necessary to understand the usage pattern, persistence, and seasonal variation of the antibiotic levels, which would in turn help in the development of appropriate policies to decrease the environmental load of antibiotics.

## Appendices

Supplementary file 

**Table 3:** Mean concentrations (µg/L) of the antimicrobials and other APIs quantified in all water samples (surface and aquifers) in 2016 and 2021

Name of the drug	Surface water			Aquifers		
	2016	2021	Fold change	2016	2021	Fold change
	Average±SD	Average±SD		Average±SD	Average±SD	
Antifungals conc (µg/L)						
Fluconazole	1.06±1.30	0.44±0.23	0.42	13.42±16.53	0.13±0.17	0.01
Ketoconazole	0.14±0.06	0.03±0.02	0.22	0.13±0.10	0.13±0.22	1.03
Itraconazole	0.02±0.01	0.34±0.25	21.63	0.01±0.01	0.22±0.17	21.71
Clotrimazole	0.25±0.13	0.02±0.01	0.10	0.2±0.20	0.01±0.01	0.02
Miconazole	1.45±3.59	0.03±0.01	0.02	0.63±0.79	0.02±0.01	0.03
Terbinafine	0.13±0.07	0±0.00	0.02	0.75±2.05	0.01±0.01	0.00
Voriconazole	0±0.00	0.01±0.01	2.09	0.01±0.00	0.01±0.00	0.94
Tinidazole	0±0.00	0±0.00	0.45	0.01±0.01	0.01±0.01	0.86
Metronidazole	0.03±0.02	0±0.00	0.00	0.06±0.05	0±0.00	0.00
Ofloxacin	1.76±1.84	0.18±0.06	0.10	4.91±7.08	0±0.00	0.00
Sparfloxacin	0±0.00	0.01±0.00	NA	0±0.00	0±0.00	NA
Antibacterial (µg/L)						
Azithromycin	0.16±0.13	0.09±0.08	0.56	0.20±0.18	0.02±0.01	0.08
Norfloxacin	0.20±0.12	0.02±0.02	0.12 ↓	0.04±0.03	0.01±0.00	0.31
Ciprofloxacin	4.88±2.94	0.02±0.02	0.00	6.13±5.23	0.01±0.01	0.00
Erythromycin	0.15±0.02	0.07±0.02	0.49	0.08±0.01	0.03±0.01	0.39
Moxifloxacin	0.16±0.02	0±0.00	0.00	0.29±0.34	0±0.00	0.00
Amoxicillin	0.27±0.18	0±0.00	0.00	0.16±0.21	0±0.00	0.00
Non-steroidal anti-inflammatory drugs (NSAIDs) (µg/L)						
Ibuprofen	1.36±0.99	0.69±0.37	0.50	0.32±0.16	0.54±0.31	1.66
Diclofenac	2.19±4.40	0.40±0.21	0.18	11.87±18.81	0.29±0.91	0.02
H2 blocker (µg/L)						
Cetrizine	3.49±8.25	0.49±0.24	0.14	2.71±6.60	0.01±0.01	0.01
Calcium channel blockers (CCBs) (µg/L)						
Amlodipine	0.07±0.07	0.01±0.02	0.15	0.08±0.09	0.01±0.01	0.10
Aminoglycosides (AG) (µg/L)						
Kanamycin	0.07±0.06	0±0.00	0.00	0.02±0.00	0±0.00	0.00
Tobramycin	0.74±0.00	4.53±0.15	6.17	0±0.00	4.46±0.13	NA
Streptomycin	0.35±0.22	0±0.00	0.00	0.25±0.06	0±0.00	0.00
Amikacin	0±0.00	1.30±0.21	NA	0.52±0.00	1.18±0.17	2.29
Netilmicin	0.20±0.06	0±0.00	0.00	0.19±0.05	0±0.00	0.00
Neomycin	1.18±0.11	0±0.00	0.00	1.05±0.32	30.22±9.98	28.68
Gentamicin	0.07±0.05	0.20±0.13	2.92	0.06±0.03	0.13±0.13	2.25

LC-MS/MS analysis of cephalosporin antibiotics

Instrumentation

LC-ESI-MS/MS experiments were performed using a triple quadrupole tandem mass spectrometer (4000 Q-Trap, AB Sciex, Foster City, CA, USA) coupled with a high-performance liquid chromatography system (HPLC, Agilent Technologies, 1260 Infinity, Santa Clara, CA, USA) consisted of a quaternary pump (G1311C), multisampler (G7167A), thermostatted column compartment (G1316A) with a variable wavelength UV detector (G1314F) and online degasser. All the tandem mass spectrometer and HPLC parameters are controlled by Analyst software, version 1.7.2 (AB Sciex, Foster City, CA, USA) and OpenLAB control panel software (Agilent Technologies, 1260 Infinity, Santa Clara, CA, USA), respectively.

Chromatographic Conditions

Chromatographic separation of cephalosporin antibiotics (cefdinir, cefalexin, cefradine, cefaclor, cefotaxime, cefadroxil, cefazolin, cefoperazone, ceftazidime, ceftiofur, cefixime, ceftriaxone, cefsulodin, cefoxitin, cephalothin, cefuroxime, cefepime, and ceftaroline fosamil) was achieved in Synergi Hydro-RP analytical column (150 x 2.0 mm, 4 µm, Phenomenex, USA) using a gradient mobile phase combination consisting of solvent A (ultra-purified water with 0.1% FA) and solvent B (methanol with 0.1% FA) pumped at a flow rate of 0.35 mL/min. The initial condition started with 10% solvent B maintained for one minute and was linearly increased to 50% (B) at five minutes and further shifted to 70% (B) by seven minutes, 90% (B) at 10 minutes and maintained till 10.5 minutes. It was equilibrated back to initial conditions at 11 minutes with a total run time of 15 minutes followed by a post-run equilibration time of two minutes. The temperature of the autosampler tray and the column oven were maintained at 10±0.8°C and 45±0.8°C, respectively. Standards and samples were injected at a volume of 2 µL for analysis.

Mass Spectrometric Conditions

Positive and negative ion detections were performed simultaneously by employing dual polarity electrospray ionization in both positive ion mode [M+H]+ and negative ion mode [M-H]- using Turbo Ion Spray source (AB Sciex, Foster City, CA, USA). Compound-dependent parameters for each analyte, such as declustering potential (DP), entrance potential (EP), collision energy (CE), and cell exit potential (CXP), were manually optimized by infusing individual standard solutions at 100 ng/mL into the ion source of the mass spectrometer at a flow rate of 10 μL/min using a Harvard pump (Harvard Company, Reno, NV, USA) connected to a Hamilton syringe (Holliston, MA, USA). Source-dependent parameters in positive ion mode were optimized and maintained as curtain gas (30 psi), collisionally activated dissociation (CAD) gas (12 psi), ion spray voltage (5500 eV), temperature (500°C), gas 1 (40 psi), and gas 2 (60 psi). The dwell time for each MRM transition was set at 25 ms. Source-dependent parameters in negative ion mode were optimized and maintained as curtain gas (30 psi), CAD gas (12 psi), ion spray voltage (-4500 eV), temperature (500°C), gas 1 (40 psi), and gas 2 (60 psi). The dwell time for each MRM transition was set at 25 ms.

Multiple reaction monitoring (MRM) mode was used for quantification of each cephalosporin antibiotic in water samples. Specific parent/product ion (m/z) transitions of all analytes and internal standard (IS) are given below, along with their respective optimized compound-dependent parameters. Sulfadimethoxine (SDM) was used as an internal standard.

**Table 4:** MRM transitions, retention time, relative retention time of 18 analytes (calculated with respect to Sulphadimethoxine, IS), and optimized ESI-MS/MS parameters

S. No	Analytes & Molecular Mass (Da)	ESI mode	Q1 mass (Da)	Q3 mass (Da)	Retention Time (min)	Relative Retention Time (min)	DP (V)	EP (V)	CE (V)	CXP (V)
1.	Cefdinir (395.0)	(+)	396.0	227.0	6.86	0.77	80	10	20	6
				126.0			80	10	42	10
2.	Cefalexin (347.1)	(+)	348.1	106.1	7.24	0.81	70	10	39	9
				158.1			70	10	14	14
3.	Cefradine (349.1)	(+)	350.1	176.1	7.68	0.86	66	10	19	5
				158.1			66	10	14	4
4.	Cefixime (453.0)	(+)	454.0	285.0	8.10	0.91	78	10	22	8
				126.0			78	10	44	11
5.	Cefaclor (367.0)	(+)	368.0	106.0	6.68	0.75	73	10	35	8
				174.0			73	10	19	4
6.	Cefotaxime (455.0)	(+)	456.0	167.0	7.52	0.84	65	10	27	4
				396.0			65	10	16	6
7.	Cefadroxil (363.1)	(+)	364.1	114.1	3.85	0.43	60	10	28	10
				208.1			60	10	16	5
8.	Cefazolin (454.0)	(+)	455.0	156.0	7.81	0.87	66	10	22	13
				323.0			66	10	16	14
9.	Ceftriaxone (554.0)	(+)	555.0	396.0	7.60	0.85	60	10	19	13
				167.0			60	10	37	15
10.	Cefsulodin (532.1)	(+)	533.1	123.1	3.10	0.35	60	10	25	7
				185.1			60	10	35	12
11.	Cefoperazone (645.1)	(+)	646.1	143.1	8.23	0.92	99	10	54	7
				290.1			99	10	31	6
12.	Cefoxitin (427.1)	(-)	426.1	156.1	8.11	0.91	-65	-10	-13	-6
				112.1			-65	-10	-24	-4
13.	Cefalothin (396.0)	(-)	395.0	180.0	9.17	1.03	-71	-10	-20	-9
				167.0			-71	-10	-9	-6
14.	Ceftazidime (546.1)	(+)	547.1	167.1	6.74	0.75	61	10	37	8
				468.1			61	10	19	21
15.	Cefuroxime (424.1)	(-)	423.1	207.1	7.67	0.86	-65	-10	-20	-11
				318.1			-65	-10	-14	-7
16.	Cefepime (480.1)	(+)	481.1	167.1	3.91	0.44	71	10	35	8
				396.1			71	10	19	16
17.	Ceftiofur (523.0)	(+)	524.0	241.0	9.26	1.04	100	10	26	6
				126.0			100	10	61	11
18.	Ceftaroline fosamil (684.0)	(+)	685.0	208.0	7.97	0.89	108	10	55	6
				262.0			108	10	50	7
19.	SDM (IS) (310.0)	(+)	311.0	156.0	8.94	-	70	10	28	7

Preparation of Calibration Standards

All cephalosporin antibiotics (salts) equivalent to their base were accurately weighed and dissolved in 50% (v/v) methanol to produce the primary stock solution at a concentration of 1 mg/mL. An amount of 1 µL (cefdinir, cefalexin, cefradine, cefaclor, cefotaxime, cefadroxil, cefazolin, cefoperazone, ceftazidime, and ceftiofur) and 10 µL (cefixime, ceftriaxone, cefsulodin, cefoxitin, cephalothin, cefuroxime, cefepime, and ceftaroline fosamil) from their respective standard stock solutions were diluted with 50% (v/v) methanol to produce 1,000 ng/mL and 10,000 ng/mL, respectively. Calibration standards were prepared by diluting this standard stock solution with blank ultra-purified water to reach concentrations ranging from 1.95 to 1000 ng/mL for cefdinir, cefalexin, cefradine, cefaclor, cefotaxime, cefadroxil, cefazolin, cefoperazone, ceftazidime, and ceftiofur and 19.5-10,000 ng/mL for cefixime, ceftriaxone, cefsulodin, cefoxitin, cephalothin, cefuroxime, cefepime, and ceftaroline fosamil, respectively.

Preparation of Surface Samples and Leachate Samples

The surface samples were aliquoted at a volume of 10 mL in 15 mL borosilicate glass tubes and subjected to lyophilization. All samples were reconstituted with 1 mL of 50% acetonitrile, vortexed for one minute, and sonicated for five minutes. The leachate samples were aliquoted at a volume of 1 mL in microcentrifuge tubes and centrifuged at 15,000 g for five minutes. An aliquot of 100 µL of reconstituted surface samples and supernatant leachate samples from each vial was mixed with 50% acetonitrile with 0.1% formic acid (FA) containing 25 ng/mL sulfadimethoxine (internal standard) followed by vortexing for one minute using a cyclomixer and then centrifugation at 15,000 g for five minutes. The resulting clear supernatant (150 μL) was subjected to cephalosporin quantification using LC-MS/MS.
